# Strongly diluted dimethyl-imidazolium chloride–alcohol solutions: solvents are structurally different but dynamic heterogeneities are similar

**DOI:** 10.1039/d1ra05633f

**Published:** 2021-11-22

**Authors:** N. A. Atamas, M. M. Lazarenko, K. S. Yablochkova, G. Taranyik

**Affiliations:** Taras Shevchenko National University of Kyiv 64, Volodymyrska Street Kyiv UA 01601 Ukraine maxs@univ.kiev.ua; International European University 42V, Akademika Hlushkova Ave Kyiv Ukraine

## Abstract

Based on the analysis of dynamic properties of ionic liquid solutions, the descriptions of diffusion mechanisms are built for dimethylimidazolium chloride (dmim^+^/Cl^−^)–alcohol solute systems and the influence of the monohydric alcohols' molecular structure on their diffusion mechanisms in dmim^+^/Cl^−^–alcohol at *T* = 400 K by molecular dynamics simulations are studied. From the analysis of radial distribution functions, MSDs, velocity autocorrelation function, and autocorrelation functions of dispersion we found that the motion of all components in IL dmim^+^/Cl^−^–alcohol (ethanol, propanol) systems at *T* = 400 K occurs in the sub-diffuse regime and that the dynamics of the dmim^+^/Cl^−^–alcohol (ethanol, propanol) systems is heterogeneous. The increase of the alkyl chain length of the alcohol molecule does not affect the motion of the ionic liquid components; instead, it increases the characteristic times describing the model representation of alcohol molecule diffusion at short and medium times, without affecting diffusion mechanisms.

## Abstract 2

The increase of the alkyl chain length of the alcohol molecule does not affect the motion of the ionic liquid components.

## Graphical abstract

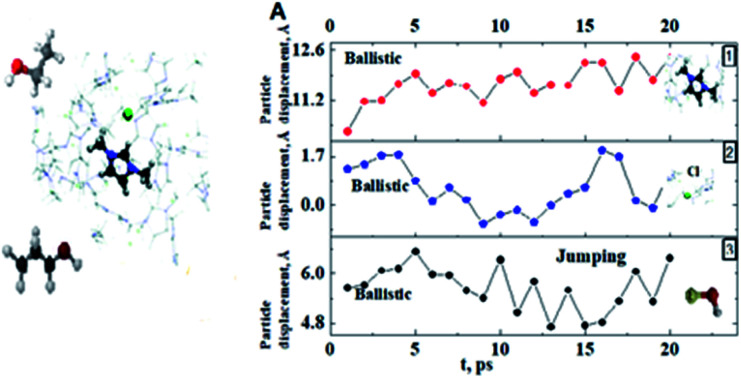

## 1. Introduction

Many unusual properties of ionic liquids (ILs) make them suitable for a wide variety of industrial applications.^1–4^ In particular, the unique combination of ionic character and high electrical conductivity of ILs opens significant practical prospects for their use as environmentally friendly solvents and catalysts.^5–10^ It's no surprise that considerable attention is drawn to the study of their physicochemical properties.^11–18^ The research in this field focuses on the establishment of a correlation between the physicochemical properties of ILs and the properties of dissolved substances and IL solutions. A variety of experimental and theoretical methods have been used to study the properties of ILs (see, for instance in ref. 19–24), however, they all share the same shortcoming: there are significant difficulties in interpreting results obtained for ionic liquids using traditional theoretical approaches for molecular liquids.^25–27^ Various experimental and theoretical studies have shown that structurally, ILs can be characterized by two or three different length scales, namely, that of common adjacency correlations.^28–30^ These approaches allow for only a partial description of the dynamic processes in ionic liquids solutions and do not allow to correctly predict the solubility processes of polar or non-polar substances in them. The processes of non-polar substances solubility in IL potentially can offer an insight into how the structure of the solute affects the motion of the IL components and the solute molecules.^31,32^ In the case of polar substances, the processes of dissolution and diffusion are much more complex not only due to the ionic nature of IL but also because of the presence of stable complexes between the solute and the ionic liquid components (as a result of Coulomb interaction). Even though the solution dynamics of ILs with polar solvents has been attracting significant attention,^33^ it would be fair to say that a theoretically justified methodology for finding a correlation between the structural characteristics of a polar substance dissolved in an IL and the rates of dissolution processes has not yet been developed.

**Fig. 1:**
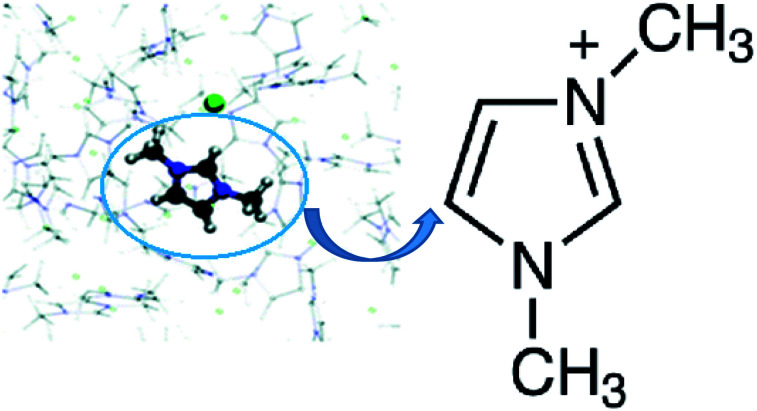
Dimethylimidazolium chloride (dmim^+^/Cl^−^).^38^

In the present work, we attempt to tackle this very issue: to find the relation between the structure of a polar substance dissolved in an IL and the process of diffusion, as well as with the dynamic heterogeneity of IL solutions. To do so, we analyze how the structure of different polar molecules belonging to the same homologous series affects the thermodynamic, structural, and kinetic properties of ionic liquid solutions. Accordingly, we present results obtained for 1,3-dimethylimidazolium chloride (dmim^+^/Cl^−^)–polar substances solutions. This ionic liquid 1,3-dimethylimidazolium chloride (dmim^+^/Cl^−^) (Fig. 1) was chosen as one of the simplest and the most commonly reported IL with an imidazolium cation, which can thus serve as a model for more complex unsymmetrical alkyl imidazolium-based IL.^34,35^ One of the classic areas of dissolution processes research is the study of motion and interactions in solutions with infinite dilution.^36,37^

This approach makes it possible to analyze the correlations of changes in the macroscopic properties of a liquid system with a change in structural characteristics depending on the polarity and structure of the solute molecules. By studying the properties of IL–dissolved polar substance systems at infinite dilution we can exclude the interactions of solute molecules with each other from consideration. Analyzing molecules of the same homological series allows one to focus on the influence of the solute structure on solubility in IL only. The conditions defined above are feasible when molecules of ethanol (C_2_H_5_OH) propanol (C_3_H_7_OH) alcohols are used as solutes. Since they belong to a monohydric alcohol homologous series and have a similar value of the dipole moment (∼1, 68 D) in liquid phase,^39,40^ they can thus serve as a simple and informative model system for studying the influence of the solute structure on the processes of polar substances dissolution in ionic liquids.

In this paper, we set out two goals: firstly, to explain how the structure of the dissolved substance affects the dynamic properties of the components of the IL solutions; secondly, to offer a viable methodology to determine the mechanisms of motion of the dissolved polar substances in ILs at different time scales. These goals are attained by performing molecular dynamics (MD) simulations of a chosen IL, and comparing the results of the simulations with the existing experimental^41–43^ and theoretical data^44,45^ obtained for the IL–polar solvent systems.

## 2. Computer experiment details

All simulations were executed using an open-source software package DL_POLY_4.05 (ref. 46) with a time step 2 fs and were carried out for systems, which consisted of 192 dmim^+^ cations, 192 Cl^−^ chlorine anions, and one dissolved substance molecule at *T* = 400 K. The following accelerated equilibration three-step strategy was used:

In step 1 an initial configuration of 192 ion pairs was manually constructed, with the ions placed at selected lattice positions within a cubic simulation box of side 33.1 Å. The density of the dmim^+^/Cl^−^ system was chosen to correspond to the experimental value of dimethylimidazolium chloride density at *T* = 400 K.^47^ The initial configuration was then equilibrated at *T* = 400 K with a constant NPT simulation for 1 × 10^6^ runs. The final configuration at *T* = 400 K was then re-equilibrated under constant NVT conditions for 1.5 × 10^6^ runs.

Then, in step 2 the final configuration at *T* = 400 K from step 1 was used as the initial configuration into which one molecule of the solute (ethanol or propanol) was added at a selected lattice position within a cubic simulation box (of side 33.1 Å). This configuration was then equilibrated at *T* = 400 K with a constant NPT simulation for 1 × 10^6^ runs. The final configuration (192 ion pairs and one molecule of the solute) at *T* = 400 K was again re-equilibrated under constant NVT conditions for 1.5 × 10^6^ runs.

Finally, in step 3 the final configuration at *T* = 400 K obtained during step 2 was used as a pre-equilibrated configuration and run under constant NVT conditions for 1.0 × 10^6^ runs as a production run with a time step 2.0 fs. The final configuration (192 ion pairs and one molecule of the solute) at *T* = 400 K was re-equilibrated under constant NVT conditions for 1.5 × 10^6^ runs with a time step 2.0 fs. When carrying out the analysis of the structural and dynamic properties of the system, a total of 2115 configurations were analyzed.

The temperature was maintained constant using “Algorithm for *p*, *T* coupling”: a Berendsen thermostat^48,49^ with incorporated SHAKE algorithm.^46^ Monitoring energy, temperature, and pressure during the time of the calculation showed that they were well established, with only small fluctuations, typical for MD simulations. Long-range electrostatic interaction was taken into account by summation, as per the Ewald method.^50^ In this work, all molecular dynamics (MD) simulations were carried out in the isothermal-isobaric (NVT) ensemble, in a cubic box with periodical boundary conditions.

The choice of the potential to describe the intermolecular interactions in the systems studied was based on the following assumptions: firstly, the structure of the ionic liquids that consists exclusively of ions is, foremost, the result of the competition between screening and packing of its components. Secondly, we have taken into account that the structure of the ionic liquid is the result of the balance between the long-range electrostatic forces (between the Cl^−^ ions and dmim^+^ cations) and the complex geometric factors arising due to the asymmetric shape of dmim^+^ cations. At the same time, the local density of the liquid is determined by the distribution of the counter-ions around certain chemical bonds and the short-range intermolecular interaction forces. The electrostatic model, reproducing the experimental crystal structures used in classical modeling of the liquid dmim^+^/Cl^−^ was described in ref. 51. It is based on the explicit atom models with partial charges and Buckingham repulsion-dispersion potentials on each atomic site. The authors of ref. 51 show that such “theoretical estimates of the lattice energy [work] sufficiently well for reasonable confidence that the intermolecular potential is adequate for simulations of model ionic liquids”. On the other hand, according to the conclusions of ref. 52, the use of the OPLS potentials to describe the interactions between the components of ILs leads to incorrect conclusions in the analysis of the liquid's local structure. Based on the results of ref. 51 and 52 we have thus chosen the Buckingham-type potentials for the fixed-point-charge force field to describe the interaction between the components of the ionic liquid. In this approach, the site–site interactions are given by the following expression:^53^1
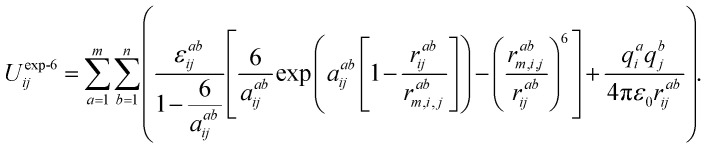


The sum of the Lennard-Jones and Coulomb components is used for the description of the interaction between the atoms of the ionic liquid components with the atoms of the dissolved substance molecules:^54^2
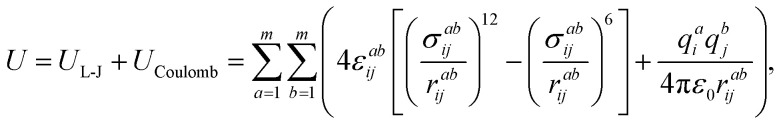
where the values of the parameters *σ*^*ab*^_*ij*_, *ε*^*ab*^_*ij*_ are L-J parameters at site *a* in a molecule *i* and site *b* in a molecule *j*, respectively, *r*^*ab*^_*ij*_ is the distance between sites *a* and *b*, *q*^*a*^_*i*_ and *q*^*b*^_*i*_ are the charges at sites *a* and *b*, respectively, and *ε*_0_ is the vacuum permittivity. The potential parameters (2)*ε*^*ab*^_*ij*_ and *σ*^*ab*^_*ij*_ for the interaction between the atoms of the IL components with the atoms of the dissolved substance molecules are calculated using the Lorentz–Berthelot combination rule. For dimethylimidazodium chloride (dmim^+^/Cl^−^) IL atomic charges and potential parameters were used according to the data from ref. 34. For the alcohol molecules, we use a triatomic model,^36^ in which a (CH_3_–CH_2_) group for ethanol or (CH_3_–CH_2_–CH_2_) group for propanol is treated as a single “effective unit-atom” atom “R” with characteristics, computed using the Lorentz–Berthelot combination rule.^54^ The geometric parameters and parameter values of the corresponding potentials for alcohols are presented.^55^

The simulation procedure provided us with the data on the dynamic and structural properties of the system for further analysis.

## 3. Results and their discussion

### 3.1. Dynamic heterogeneous properties in IL–alcohols solution

**Fig. 2:**
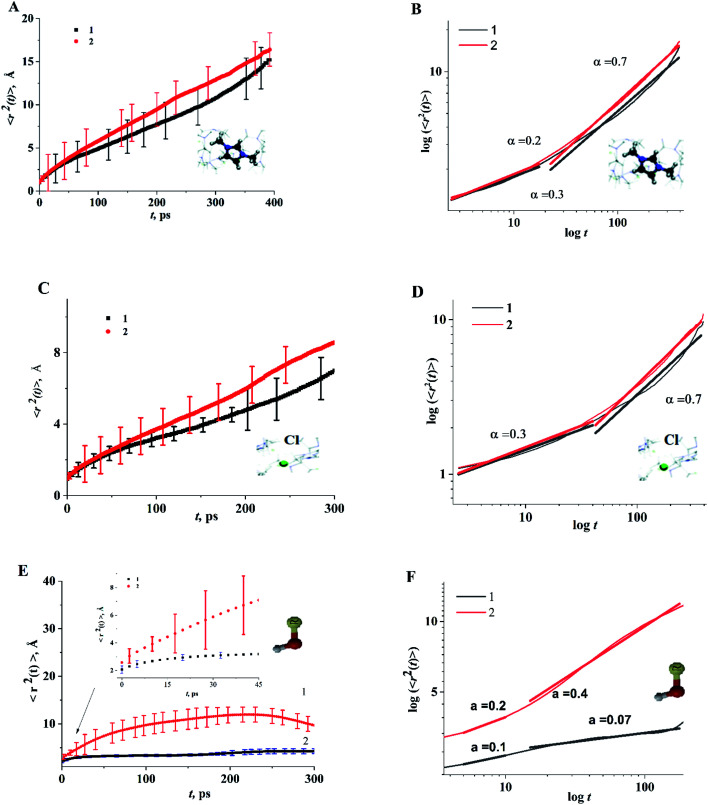
Time dependence of function 〈*r*^2^(*t*)〉 of the center of mass of cation dmim^+^ (A and B), anions Cl^−^ (C and D), alcohol molecules (E and F) in the dmim^+^/Cl^−^–alcohol systems at *T* = 400 K (1 – ethanol; 2 – propanol).

As structural heterogeneity of ionic liquids is closely related to changes in the dynamic processes in them,^56–59^ we conducted a study of the dynamic heterogeneity of the systems under study, determined by changes in diffusion processes with time. To determine the time intervals within which there are no changes in diffusion mechanisms, it is necessary to analyze the trajectories of motion of the solute (ethanol and propanol molecules) and ionic liquid components on short-, medium- and long-time scales using the mean squared displacement, the MSD. The MSD 〈*r*^2^(*t*)〉 (Fig. 2A–E) is defined as 
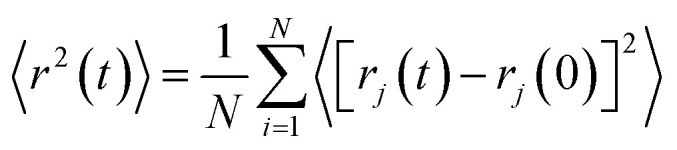
, where the sum runs over the total number of particles *N*, and the brackets denote a suitable ensemble average.^60^ The qualitative characteristic of the dynamic heterogeneities in the liquid system can be obtained by determining the diffusion mechanisms in it. This can be achieved by analyzing parameter *α* in log〈*r*^2^(*t*)〉 ∼ *a* log *t*, whose values reveal, how the diffusion of particles deviates from the Brownian diffusion.^57^

Based on data shown in Fig. 2 (*α* < 1) and according to the conclusions of ref. 56, IL–alcohol components move in a slowed sub-diffuse regime, similar to that of particles in super-cooled liquids.^27,61^ The values of *α* are notably lower for alcohol molecules than for the components of dmim^+^/Cl. MSDs analysis (Fig. 2E) shows that the heavier and bigger molecule of propanol moves faster than the ethanol molecule, and the statistical errors of the MSD calculations (determined using methods described in ref. 62 and 63) for the propanol molecule are higher than the errors of the MSD calculations for the ethanol molecule.

When the alcohol molecule dissolves in dmim^+^/Cl, the local structure of the IL changes and it can form hydrogen bonds with the components of dmim^+^/Cl^−^. The local structure established can act as “defects”, leading to the slowed sub-diffuse regime of all components in the dmim^+^/Cl^−^–alcohol system. The existence of a plateau in the *t*-dependence of the MSD for propanol at *t* > 30 ps supports this notion. The slowing down of the ethanol molecule in dmim^+^/Cl^−^ is observed at *t* > 10 ps. The changes in the values of *α* (Fig. 2) at different time scales and the change in the solute's time dependence of motion (Fig. 4) indicate the presence of different diffusion components in the dmim^+^/Cl^−^–alcohol system at different time scales. In other words, they support the assumption of the heterogeneity of the dmim^+^/Cl^−^–alcohol system's components dynamics.

To achieve this, we followed the methodology proposed by the authors of ref. 64 and 65. The following was taken into account: since the IL dmim^+^/Cl^−^ has a melting point *T* = 399 K,^66^ it should be expected that at *T* = 400 K the interaction energy between the components of the IL would be quite large and the diffusion processes would be determined just as in the electrolyte salt close to the melting point. According to ref. 64 and 65 at a temperature close to the melting point a liquid has a solid-like oscillation spectrum at high frequencies. In this case, phonon type oscillations exist in liquids at frequencies exceeding the value of the relaxation time τ*: *ω* > ω* = 2π/τ*. When the relaxation time τ* becomes comparable to the minimum time *τ*_0_ of vibrations, the most short-wavelength transverse oscillations disappear from the liquid's oscillation spectrum. At the same time (τ* < *τ*_0_), according to ref. 64, the characteristic time between jumps of a particle over distances comparable to inter-particle distance significantly exceeds the shortest time of the particle's oscillation period *τ*_0_ = 2π/*ω*_0_, where *ω*_0_ is the maximum frequency of acoustic disturbances in the system (of the Debye frequency order) equals the relaxation time τ*. However, it is difficult to separate the vibrational mechanisms from the vibrational-jumping mechanisms of particle diffusion in the IL dmim^+^/Cl^−^, as the particle makes one or two oscillations between jumps. The analysis of the autocorrelation functions of dispersion *F*_s_(*q*,*t*) helps us clarify this issue and determine the values of the corresponding times at which the diffusion mechanisms of the system change:^65^3
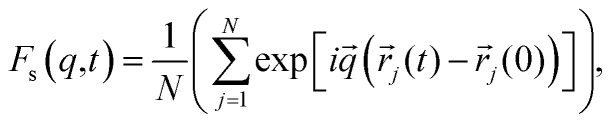
where *r⃑*_*j*_ is the radius vector of the *j*-particle.

**Fig. 3:**
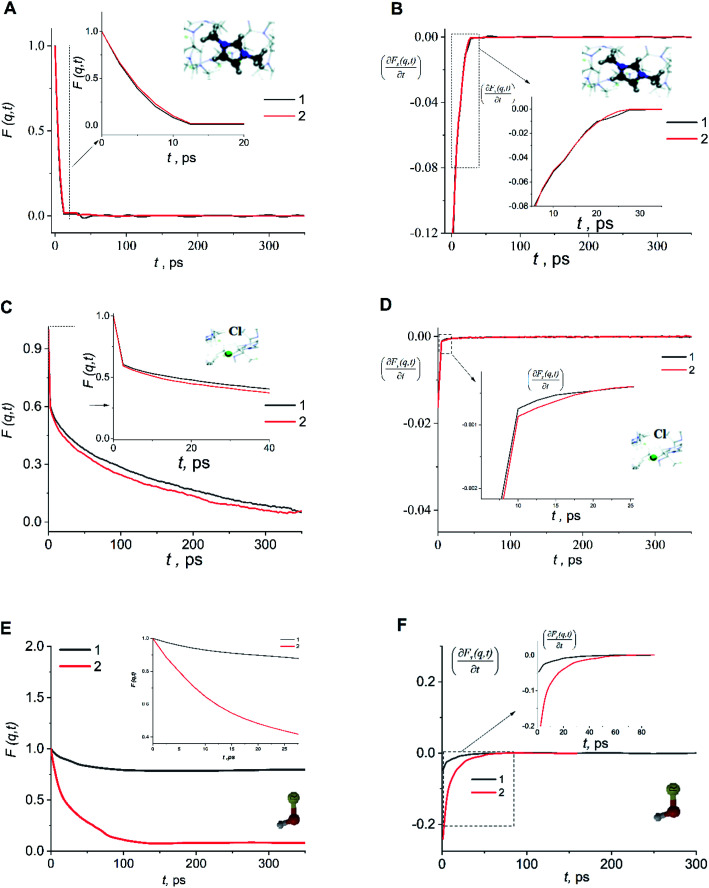
Autocorrelation functions of dispersion *F*_s_(*q*,*t*) and first time derivatives of autocorrelation functions of dispersion 
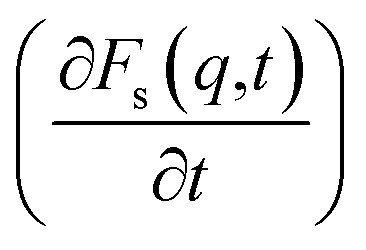
 for dmim^+^ (A and B), anions Cl^−^ (C and D) and alcohols (E and F) in the dmim^+^/Cl^−^–alcohol systems (1 – ethanol; 2 – propanol) at *T* = 400 K.

**Fig. 4:**
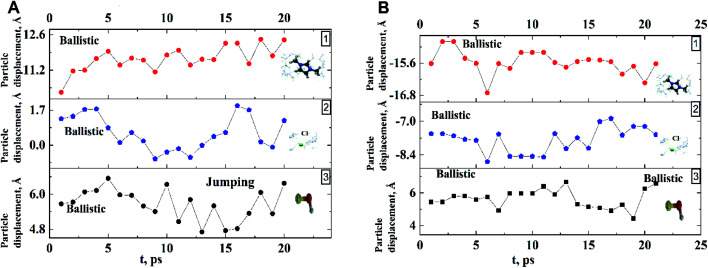
Examples of the averaged trajectories of components (along *x*-axis) of dmim^+^/Cl^−^–alcohol systems ((A) dmim^+^/Cl^−^–ethanol: 1 – dmim^+^, 2 – Cl^−^, 3 – ethanol), (B) dmim^+^/Cl^−^–propanol: 1 – dmim^+^, 2 – Cl^−^, 3 – propanol) at *T* = 400 K.

An analysis of the autocorrelation functions of dispersion *F*_s_(*q*,*t*) (Fig. 3A, C and E) and their derivatives 
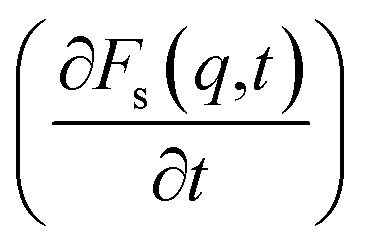
 (Fig. 3B, D and F) for the dmim^+^/Cl^−^ components, as well as for solute molecules, can be used to separate the vibrational, collision, and ballistic diffusion regimes of the liquid components.^65^ Special attention should be given to the analysis of 
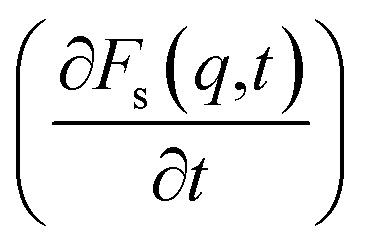
 behaviour, as the inflection points of the corresponding curves allow determining the times of diffusion regime change in the system. The functions *F*_s_(*q*,*t*) (Fig. 3A and C) of dmim^+^ of the dmim^+^/Cl^−^–alcohol systems are identical at times *t* < 12 ps. The functions *F*_s_(*q*,*t*) (Fig. 3A and C) of Cl^−^ of the dmim^+^/Cl^−^–alcohol systems, on the other hand, are identical at times *t* < 5 ps. The *t*-dependence of the *F*_s_(*q*,*t*) functions of dmim^+^ and Cl^−^ indicates that the diffusion mechanisms of dmim^+^ and Cl^−^ in them are the same at times *t* < 5 ps and, therefore, the components of the IL dmim^+^/Cl^−^ can move as part of joint complexes. The obtained data confirm the assumptions of the authors of ref. 67 and 68 about the existence of different clusters in the ILs formed by the components of the IL and an independent “swimming” of Cl^−^ anions in dmim^+^/Cl^−^. As can be seen by the derivatives 
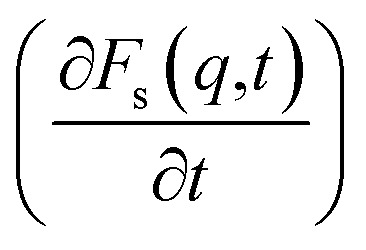
 (Fig. 4B and D), the change in the diffusion mechanism of the dmim^+^ cations is observed at *t* ∼ 18 ps, whereas the change in diffusion mechanism of the Cl^−^ anions is observed at *t* ∼ 10 ps. The result shows a possible independent movement of Cl^−^ anions in the dmim^+^/Cl^−^ and correlates with the experimental data of ref. 69.

Analysis of the function 
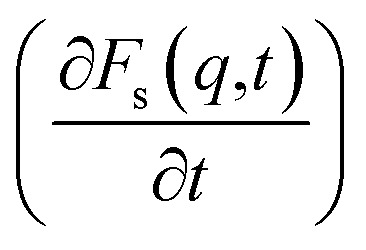
 for alcohol shows that changes in the ethanol diffusion mechanism occur initially at *t* ∼ 10 ps for ethanol and *t* ∼ 30 ps for propanol. Analysis of the motion of particles along the *x*-axis (Fig. 4) at short times for *t* < 30 ps clearly demonstrates the change in the mechanisms of the diffusion of the system's components. In particular, the nature of the time dependence of the trajectories of the components of dmim^+^/Cl^−^–alcohol systems allows us to determine the mechanisms of diffusion.^70^ In Fig. 4A, fragments of trajectories (along the *x*-axis) show that ballistic collisions dominate in the motion of all components of the dmim^+^/Cl^−^–ethanol system at short times *t* < 10 ps. With increasing time at *t* > 10 ps, the motion of ionic liquid components in the dmim^+^/Cl^−^–ethanol system does not change; this motion is due to the ballistic collisions. The motion of the ethanol molecule at *t* > 10 ps can be described in the framework of the jump-diffusion model: the jumps from one equilibrium position into another. The motion of all components in the dmim^+^/Cl^−^–propanol (Fig. 4B) system at *t* < 30 ps is dominated by ballistic collisions.

The difference in the motion mechanisms of the dissolved matter's molecules at short times, in our opinion, can be attributed to the structure of dissolved alcohol molecules and their ability to form co-complexes with the components of the ionic liquid.

At times 50 ps < *t* < 300 ps, the derivatives 
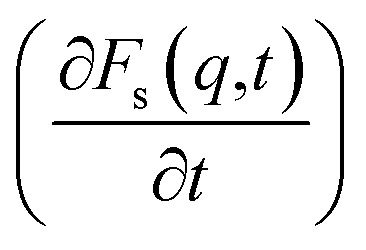
 for ethanol and propanol alcohols have almost identical behaviour, so the diffusion mechanisms of these molecules are the same.

### 3.2. Determination of characteristic times in models of diffusion mechanisms of alcohols in IL solution

Analysis of the velocity autocorrelation function (VAF) allows us to determine the time of rotation of the alcohol molecule in its equilibrium position. The velocity autocorrelation function (VAF), which determines the velocity of a tagged particle moving through a fluid in the *x*-direction is defined as:^72^4
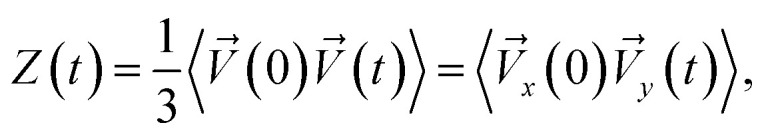
*V⃑*(0) – is a measure of the projection of the particle velocity onto its initial value, averaged over initial conditions. At times long enough (compared to any microscopic relaxation times) the initial and final velocities are completely uncorrelated. The position of the first zero of the VAFs *Z*(*t*) represents the average collision time *t*_coll_ due to the “cage” effect, typical for the liquid phase.

**Fig. 5:**
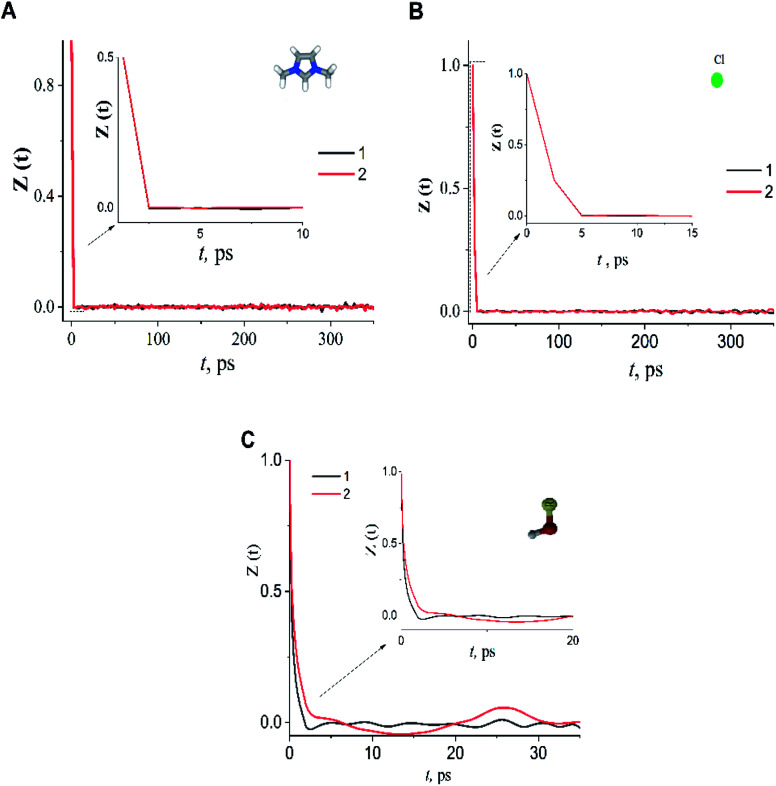
The velocity autocorrelation function *Z*(*t*) of dmim^+^ (A), Cl^−^ (B) and alcohols (C) in dmim^+^/Cl^−^–ethanol system (1), dmim^+^/Cl^−^– propanol system (2) at *T* = 400 K.

In the case of the dmim^+^/Cl^−^–alcohols systems at *T* = 400 K, the VAFs *Z*(*t*) functions (Fig. 5) show an oscillatory behaviour similar to that of rigid-ion models of inorganic molten salts.^73,74^ Analysis of our calculated VAFs *Z*(*t*) (Fig. 5) show that *Z*(*t*) of the cation dmim^+^ and anion Cl^−^ decay quickly, reaching a zero at about *t*^dmim^_coll_ = ∼2.0 ps *t*^Cl^_coll_ ∼ 5.0 ps. Note, that this agrees with the results of ILs simulations outlined in ref. 67. The VAFs *Z*(*t*) of the ethanol and propanol behave differently: VAF *Z*(*t*) of the ethanol decays to zero at *t*^ethanol^_coll_ ∼ 3.0 ps and reaches a negative asymptotic plateau at about ∼5.0 ps; whereas VAF *Z*(*t*) of the propanol decays to zero at *t*^propanol^_coll_ ∼ 8.0 ps.

Taking into account the data obtained, the diffusion of alcohol molecules in the IL can be represented within the framework of the “Swiss cheese” model with different characteristics times: a free space “cavity” is formed around the alcohol molecule in the equilibrium position as a result of ballistic collisions with the IL components, leading to a change in the local structure of the liquid. The subsequent motion of the alcohol molecules can be represented within the framework of the jump-diffusion model by the interparticle distance to the next equilibrium position. While in the equilibrium position, the alcohol molecules can also undergo vibrational–rotational motion.^75^

### 3.3. Diffusion models of a highly diluted solution dmim^+^/Cl^−^–alcohols systems

According to ref. 76, IL dimethylimidazodium chloride (dmim^+^/Cl^−^) can be treated as a mixture of large (dmim^+^) and small particles (Cl^−^) with a mass ratio of *α*_m_ = *M*_large_/*M*_small_ = *M*_dmim_/*M*_Cl_ = 2.7. Additionally, according to the conclusions of ref. 71, the structural rearrangement in the dmim^+^/Cl^−^ system is determined by the relaxation processes, which can be described within the framework of the short-time diffusion model. Based on the analysis of the MSD, VAFs *Z*(*t*) and 
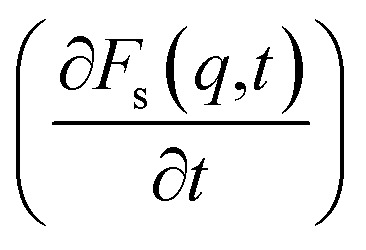
 time dependences (Fig. 2–5), we can assume that at times *t* < 10 ps, the local structure dmim^+^/Cl^−^ is rearranged due to ballistic collisions between particles. Based on the data presented above, we can assume that at *t* < 10 ps the diffusion of the ethanol molecule and at *t* < 15 ps (in the case of the dmim^+^/Cl^−^–propanol molecule) diffusion in the dmim^+^/Cl^−^ is the result of inelastic collisions with the IL components and jumps in the interparticle distance, which is a prerequisite for the local liquid structure change due to the restructuring of the hydrogen bond network in the system. At the *t* > 10 ps for the dmim^+^/Cl^−^–ethanol system and at the *t* > 15 ps for the dmim^+^/Cl^−^–propanol system, according to Frenkel theory,^72^ the diffusion processes in dmim^+^/Cl^−^–alcohol systems at *T* = 400 K (just as in the electrolyte salt) would be determined by the activation jumps of the components (*via* the so-called, vibrational-jumping mechanism). In the intervals between such jumps, the dmim^+^/Cl^−^ components like electrolyte salts can also oscillate around the equilibrium position. All dmim^+^/Cl^−^–alcohol systems under consideration are harmonic oscillatory systems with rare particle jumps. The diffusion mechanisms of dmim^+^ cations at these times are the same: cations dmim^+^ can rotate in the equilibrium position both as parts of joint complexes and individually. Concurrently, Cl^−^ anions can move both as a part of complexes with cations and independently. At time intervals (10 ps < *t* < 40 ps) diffusion mechanisms of the dmim^+^ cations and Cl^−^ anions in the dmim^+^/Cl^−^ system remain unchanged. At *t* > 10 ps (Fig. 3–5), the diffusion mechanisms of ethanol and propanol are different. In this case, the relaxation time *τ*_0_ can be represented as the average jump time *τ* to the inter-particle distance:^65^5
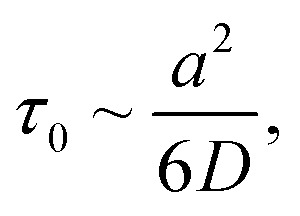
where *a* – is the average inter-particle distance, the value of which is related to the size of the free space formed around the dissolved substance, *D* – self-diffusion coefficient. The average inter-particle distance *a* can be obtained from the analysis of the radial distribution functions RDF *G*_*XY*_(*r*), which gives the probability of finding particles of type *y* near particles of type *x*:^54^6
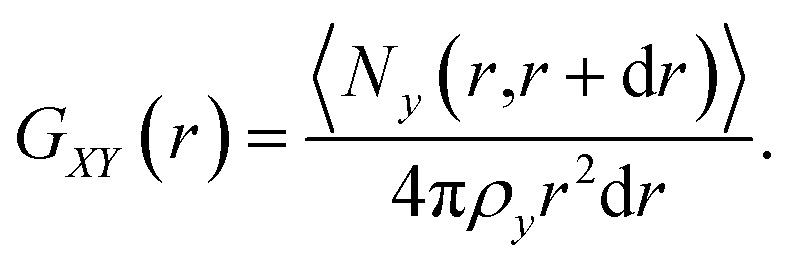


In this equation, the numerator describes the average number of particles *y* in a spherical layer (*r*, *r* + d*r*), and the denominator normalizes the distribution so that *G*_*XY*_(*r*) = 1 at *N*_*y*_ = *ρ*_*y*_, where *ρ*_*y*_ is the density. The average values of the short-time self-diffusion coefficient *D* can be obtained by integrating the VAF *Z*(*t*):^24^7
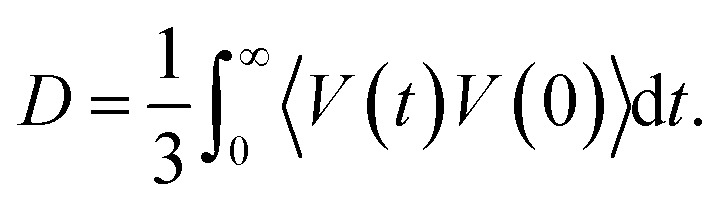


Near the melting point, the characteristic values of the self-diffusion coefficient correspond to the times of relaxation τ*, comparable with the times of one vibration *τ*_0_.

In our previous paper ref. 37 we analyzed the local structure of the dmim^+^/Cl^−^–alcohol systems at *T* = 400 K and have shown that the average distance between dmim^+^ cations in such systems is ∼3.1 Å and the average distance between Cl^−^ anions is about ∼5.9 Å. In ref. 77 we have also demonstrated that in case of interaction between the Cl^−^ anion and the hydrogen atoms of the dmim^+^ cation hydrogen bonds with lengths of ∼2.8 Å are formed.

**Fig. 6:**
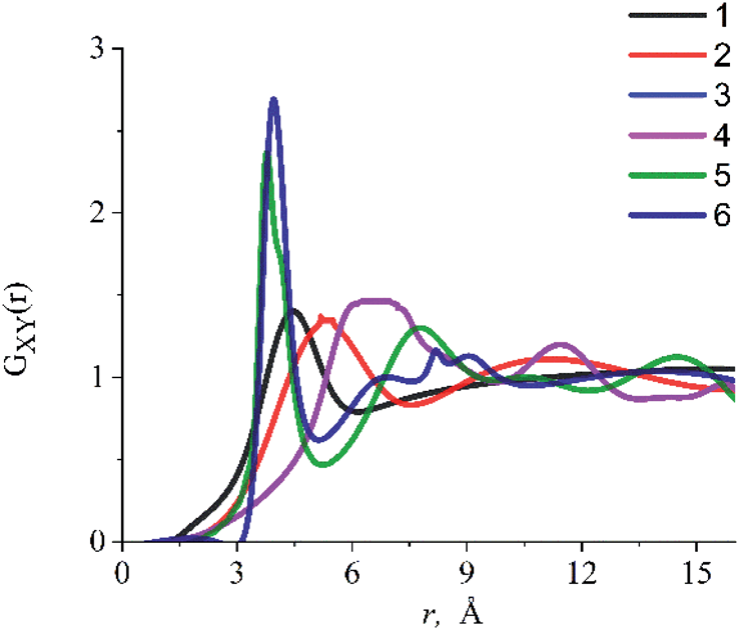
Radial distribution functions for the (1) O^eth^–C^dmim^+^^, (2) O^prop^–C^dmim^+^^, (3) H^eth^–C^dmim^+^^, (4) H^prop^–C^dmim^+^^, (5) “unit-atom” of ethanol (C_2_H_5_)^eth^–Cl^−^, (6) “unit-atom” of propanol (C_3_H_7_)^prop^–Cl^−^. Each radial distribution function is calculated from the center of mass of the imidazolium ring C^dmim^+^^.

The RDFs obtained in the presented work were used to determine the inter-particle distance between the dissolved particles and IL components in the systems under study. RDFs analysis (Fig. 6) shows that there is a strong interaction between the oxygen atom of the alcohol molecule (O^eth^, O^prop^) and the center of mass of the dmim^+^ cation (C^dmim^+^^), due to which the ethanol molecule is located at ∼4.8 Å from the dmim^+^ cation and the propanol molecule is ∼5.3 Å from the dmim^+^ cation. We also found that OH^−^ group of alcohols and Cl^−^ can form a complex with Cl^−^ (length ∼2.8 Å). Taking into account our results and considering that, within the framework of the selected model representations, at the first approximation, the ethanol molecule can be considered as a sphere with an effective van der Waals radius *R*_vdW_ = 1.9 Å,^78^ we can assume that when it is dissolved in dmim^+^/Cl^−^, free space “cavity” with the maximum radius of ∼5.9 Å is formed and the nearest inter-particle distance is *a* ∼ 7.8 Å. Upon dissolution of propanol in dmim^+^/Cl^−^ (*R*_vdW_ = 2.0 Å),^78^ free space of maximum radius ∼ 6.3 Å is formed around it, and the closest inter-particle distance, in this case, is *a* ∼ 8.3 Å.

The average value of self-diffusion coefficient (7) for dmim^+^ cations is different in ethanol and propanol-systems: *D*^et^_dmim_ = 4.0 × 10^−11^ m^2^ s^−1^ in dmim^+^/Cl^−^–ethanol and *D*^prop^_dmim_ = 5.0 × 10^−11^ m^2^ s^−1^ in dmim^+^/Cl^−^–propanol. The diffusion coefficients for Cl^−^ anions in the systems under consideration are identical *D*^et^_Cl_ = *D*^prop^_Cl_ = 3.0 × 10^−11^ m^2^ s^−1^. These numbers are in good agreement with the values obtained,^79^*D*_dmim_ = 4.23 × 10^−11^ m^2^ s^−1^ and *D*_Cl_ = 2.3 × 10^−11^ m^2^ s^−1^. The self-diffusion coefficients are larger for the dmim^+^ cation in all the alcohol solvents studied, in an apparent contradiction to its larger size and mass, compared to the anion Cl^−^. The fact that the lighter anions have a smaller self-diffusion coefficient has also been observed experimentally and reported in ref. 80.

We can thus infer that an increase in the mass and the complexity of the structure of the alcohol molecule leads to an increase in the diffusion coefficient for dmim^+^/Cl^−^: the self-diffusion coefficient of ethanol in dmim^+^/Cl^−^ is *D*_et_ = 5 × 10^−11^ m^2^ s^−1^, the self-diffusion coefficient of propanol in dmim^+^/Cl^−^ is *D*_prop_ = 7 × 10^−11^ m^2^ s^−1^. The difference in the self-diffusion coefficients of alcohol molecules in dmim^+^/Cl^−^ is caused by both a difference in the molecules' structure and a difference in the mechanisms of interaction between alcohol molecules and IL components at short times *t* < 10 ps. Then, according to ref. 64, the jump time of the alcohol molecules in dmim^+^/Cl^−^ to the inter-particle distance, which coincides with the relaxation time of the system at *T* = 400 K, is ∼202 × 10^−11^ s for ethanol and ∼187 × 10^−11^ s for propanol. The present results thus demonstrate that to describe diffusion in the systems under consideration at times *t* < 300 ps at least two models could be applied. It also indicates that the mechanisms of diffusion, as well as the dynamic heterogeneity of the system changes, which correlates both with the experimental data obtained *via* neutron spin-echo and confocal microscopy methods,^80,81^ and with computer simulations.^82,83^

## 4. Conclusions

The paper presents the influence of monohydric alcohol molecules with the same dipole moment on the dynamic characteristics of the IL dmim^+^/Cl^−^–alcohol (ethanol, propanol) systems at *T* = 400 K. The motion of alcohol molecules dissolved in ILs is associated with the change in the local structure of the ILs around them. This motion has two main stages: first, the free space is formed around the solute molecule, then the hydrogen bonds form between the molecules of the dissolved substance and the components of IL. Based on the MSD analysis we show that the motion of all components in the dmim^+^/Cl^−^–alcohol (ethanol, propanol) systems at *T* = 400 K takes place in a slowed sub-diffuse regime and the motion mechanisms of the system's components change over time, *i.e.* their dynamics is heterogeneous. As the structural rearrangement in the dmim^+^/Cl^−^ system is determined by the relaxation processes, which can be described within the framework of the short-time diffusion model, we offer the model representations for the diffusion mechanisms for the IL components studied at small times:

At *t* < 10 ps (dmim^+^/Cl^−^–ethanol system) and *t* < 30 ps (dmim^+^/Cl^−^–propanol system) the diffusion of the alcohol molecule in dmim^+^/Cl^−^ occurs due to inelastic ballistic collisions with IL components. At this time scale, the interval between the collisions between the alcohol molecules and the IL components is *t*^ethanol^_coll_ ∼ 3.0 ps for the ethanol and *t*^propanol^_coll_ ∼ 8.0 ps for the propanol molecules. The diffusion of alcohol molecules can be described in terms of the ballistic model;

At *t* > 10 ps (dmim^+^/Cl^−^–ethanol system) and *t* > 30 ps (dmim^+^/Cl^−^–propanol system) the diffusion of the alcohol molecule in dmim^+^/Cl^−^ is due to inelastic collisions with the components of dmim^+^/Cl^−^ and jumps to inter-particle distances with a characteristic jump time of ∼20.2 ps for ethanol and ∼18.7 ps for propanol. Between the jumps in their equilibrium position, alcohol molecules make one or two oscillations.

In summary, the increase of the alkyl chain length of the alcohol molecule with the same dipole moment value that is dissolved in an ionic liquid does not affect the motion of the ionic liquid components; instead, it increases the characteristic times describing the model representation of alcohol molecules diffusion at short and medium times, without affecting diffusion mechanisms.

## Conflicts of interest

There are no conflicts to declare.

## Supplementary Material
